# AMIC for Focal Osteochondral Defect of the Talar Shoulder

**DOI:** 10.3390/life10120328

**Published:** 2020-12-05

**Authors:** Christian Götze, Christian Nieder, Hanna Felder, Filippo Migliorini

**Affiliations:** 1 Department of Orthopaedic Surgery, Auguste-Viktoria Clinic, Ruhr University Bochum, 32545 Bad Oeynhausen, Germany; Christian.Goetze@muehlenkreiskliniken.de (C.G.); christian.nieder@muehlenkreiskliniken.de (C.N.); Hanna.Felder@ruhr-uni-bochum.de (H.F.); 2 Department of Orthopaedics and Trauma Surgery, University Clinic Aachen, RWTH Aachen University Clinic, 52064 Aachen, Germany

**Keywords:** talus, osteochondral defects, osteochondrosis dissecans, AMIC

## Abstract

Background: The management of a focal osteochondral lesion of the talus (OLT) is challenging. Evidence concerning the role of the autologous matrix-induced chondrogenesis (AMIC) procedure in patients with focal OLT is promising. The purpose of the present study was to investigate clinical outcomes and radiographic findings of the AMIC technique for focal unipolar OLT. Material and Methods: The present study was performed according to the Strengthening the Reporting of Observational Studies in Epidemiology (STROBE) guidelines. Twenty-four patients who underwent AMIC for focal OLT were prospectively recruited at our institution. All the surgeries were performed by two experienced surgeons through malleolar osteotomy and autologous cancellous bone grafting. The outcomes of interest were the American orthopedic foot and ankle score (AOFAS), the foot-function index (FFI), and the magnetic resonance observation of cartilage repair tissue (MOCART). Surgical duration, hospitalization length, and complications were also collected. Results: 24 patients were included in the present study. The mean follow-up was 25.17 ± 13.1 months. The mean age of the patients at surgery was 46.75 ± 15.2 years, the mean BMI 26.92 ± 5.7 kg/m^2^, and 50% (12 of 24) of patients were female. The right ankle was involved in 62.5% (15 of 24) of patients. The mean defect size was 6.95 ± 2.9 mm^2^. The mean surgical duration was 112 ± 20 min while the mean hospitalization 5.58 ± 1.7 days. At last follow-up, the AOFAS increased by 27.8 points (*p* < 0.0001), while the FFI reduced by 25.3 points (*p* < 0.0001) and the MOCART score increased by 28.33 points (*p* < 0.0001). No complications were observed. Conclusion: The AMIC procedure for focal osteochondral defects of the talar shoulder is feasible and reliable at midterm follow-up.

## 1. Introduction

Osteochondral lesions of the talus (OLT) are circumscribed defects of the articular layer and its underlying subchondral bone. Their etiology remains not fully understood, although an association with post-traumatic instability of the ankle has been demonstrated [1]. Other etiological factors, such as genetic, metabolic, ischemic, endocrine, and vascular, have been described [2,3,4]. Symptoms of OLT are unpredictable: it can be incidentally diagnosed in asymptomatic patients or can cause severe pain and limitation of daily activities [5]. 

Given the limited cartilage intrinsic repair ability, these patients are challenging to treat [6,7]. Conservative therapies demonstrated limited efficacy [8,9]. Therefore, a surgical approach is usually recommended for OLT [10,11,12]. Autologous matrix-induced chondrogenesis (AMIC) is a single-stage surgical procedure that exploits the potential of bone-marrow-derived mesenchymal stem cells [13,14]. AMIC consists in curettage and debridement of non-viable surrounding tissue followed by microfractures of the subchondral bone. The defect is afterwards covered using a collagen I/III membrane. Current literature lacks prospective trials analyzing AMIC for focal OLT at midterm follow-up. 

The purpose of the present study was to assess clinical outcomes and radiographic findings of AMIC for the management of focal OLT. A multivariate analysis was conducted to evaluate potential prognostic factors. We hypothesized that AMIC yields reliable results with a low rate of complications at 24-months follow-up. 

## 2. Material and Methods

### 2.1. Study Design 

The present study was performed according to the Strengthening the Reporting of Observational Studies in Epidemiology, the STROBE statement [15]. All procedures reported in the present investigation were approved by the Ethics Committee of the Medical Faculty of the Ruhr University of Bochum, Germany (EK 2017-164). This study was conducted according to the principles expressed in the Declaration of Helsinki. All patients were able to understand the nature of their treatment and provided written consent to use their clinical and imaging data for research purposes. 

### 2.2. Eligibility Criteria

Data from patients that underwent AMIC for focal osteochondral defects of the talar shoulder were collected in a prospective fashion. Patients were recruited at the Auguste Viktoria Clinic in Bad Oeynhausen, Germany, in the period 2013 to 2017. The inclusion criteria were: (1) symptomatic chronic ankle pain, (2) previous failed conservative treatments, (3) evidence of osteochondral defect of the talar shoulder on MR, (4) focal defect, (5) traumatic onset, (6) intact surrounding cartilage, (7) lesion > 1.0 mm^2^, and (8) patients aged 18 to 55 years. The exclusion criteria were: (1) metabolic arthropathies, (2) kissing lesions, (3) large non-reconstructable defects, (4) uncorrectable axial misalignments, (5) chronic inflammatory systemic disease, (6) body mass index, BMI > 30 Kg/m^2^, and (7) bilateral ailments.

### 2.3. Surgical Technique

All the surgeries were performed in the same fashion by two experienced surgeons (CG and CN) according to a previous report [16]. Briefly, lesions were approached through a malleolar osteotomy. The line of osteotomy was performed at the junction of the plafond to obtain adequate exposure of the lesion. After identification of the OLT, this was debrided of the non-viable surrounding bone tissue. The remaining defect was filled with autologous cancellous bone graft harvested from the osteotomy site or from the homolateral iliac crest. Debridement and curettage of the non-viable border of the chondral tissue surrounding the lesion was then performed until viable shoulder cartilage was reached. An aluminum template is then used to size the defect. A type I/III collagen membrane (Chondro-Gide^®^, Geistlich Pharma AG, Wolhusen, Switzerland) was trimmed according to the aluminum template to be slightly undersized in relation to the defect to avoid displacement and hydrated in a saline solution. Microfractures of 4 mm depth were performed into the defect using 1.2 or 1.4 mm K-wire under constant irrigation to avoid thermal necrosis. The membrane was placed into the lesion and secured with fibrin glue. The stable position of the membrane was checked by flexing and extending the ankle. Osteotomy was fixed with two malleolar screws that were inserted though predrilled holes and the wound sutured. Non-weightbearing for the first six weeks in a vacopedis boots was required. A passive range of motion exercises were started two days after surgery, after the first change of dressing, starting with a maximal range of motion of 20° and manual lymphatic drainage massage took place for at least six weeks. Afterwards, an intensive rehabilitation program started, with increasing weight-loading to full weight-bearing as tolerated and strengthening of the lower leg muscles and ankle joint stability by proprioception training. 

### 2.4. Outcomes of Interest

On admission, the following data were recorded: age, gender, side, size of defect, BMI, and duration of symptoms. An MR scan was conducted to assess the magnetic resonance observation of cartilage repair tissue (MOCART) [17]. Further, the foot-function index (FFI) [18], and the subscale hindfoot of the American Orthopedic Foot and Ankle Score (AOFAS) [19] were administered. These aforementioned scores were administered preoperatively and at last follow-up. Surgical duration and length of the hospital stay were recorded, as were complications. 

### 2.5. Statistical Analysis

All statistical analyses were performed by one author (FM) who had not been involved with the clinical management of the patients using the software STATA/MP 14.1 (StataCorp, College Station, TX, USA). Mean difference (MD) effect measure was adopted for continuous variable. The unpaired *t*-test was used to assess significance, with *p* values < 0.05 considered statistically significant. A multivariate analysis was performed to assess associations between data at baseline (age, gender, side, size of defect, BMI, duration of symptoms, surgical duration, hospitalization length, MOCART, AOFAS, and FFI) and these data at last follow-up (MOCART, AOFAS, and FFI). A multiple linear model regression analysis through the Pearson product-moment correlation coefficient (r) was used. The Cauchy-Schwarz formula was used for inequality: +1 was considered as positive linear correlation, while −1 was considered a negative one. Values of 0.1 < |r| < 0.3, 0.3 < |r| < 0.5, and |r| > 0.5 were considered to have weak, moderate, and strong correlation, respectively. The overall significance was performed through the χ^2^ test, with values of *p* < 0.05 considered statistically significant. 

## 3. Results

### 3.1. Recruitment Process

**Figure 1:**
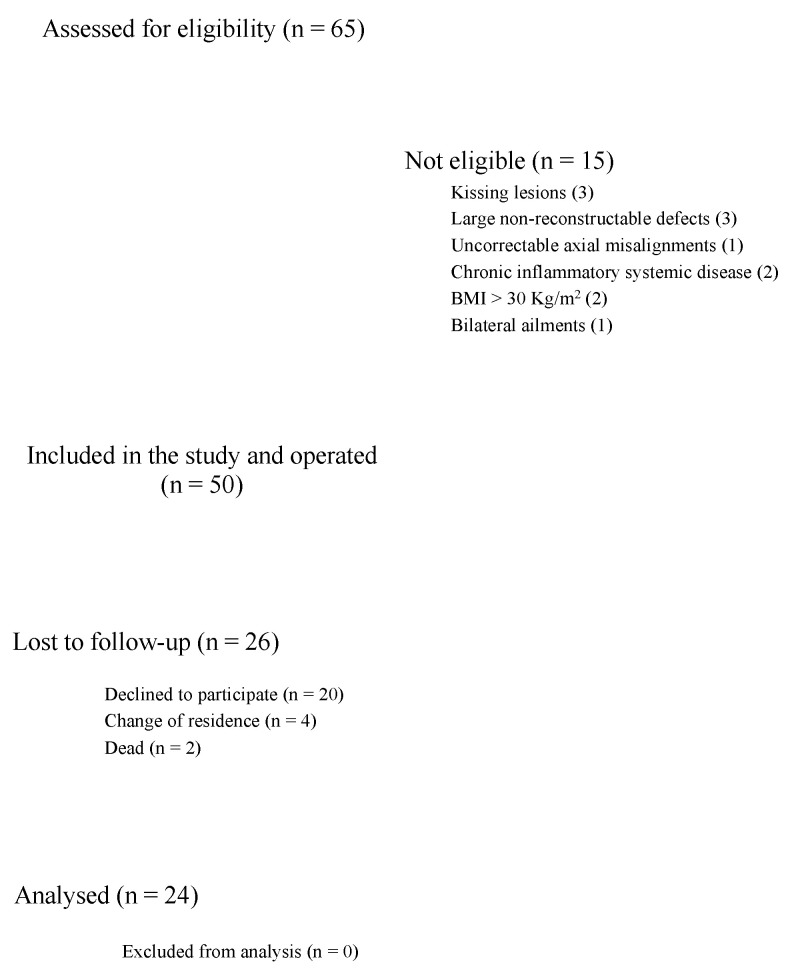
Diagram of the recruitment process.

A total of 65 patients with OLT underwent AMIC during the study period. Fifteen patients were not eligible-kissing lesions (3), large non-reconstructable defects (3), uncorrectable axial misalignments (1), chronic inflammatory systemic disease (2), BMI > 30 Kg/m^2^ (2), and bilateral ailments (1). A total of 50 patients underwent the index procedure. Patients did not take part in the study because of the following reasons: change of residence (N = 4), died (N = 2), did not wish to participate in the study (N = 20). Finally, 24 patients were included in the present study. The diagram of the recruitment process is shown in Figure 1.

### 3.2. Patient Demographics

The mean follow-up was 25.17 ± 13.1 months. The mean duration of symptoms before the surgical intervention was 24.77 ± 32.1 months. The mean age of the patients at surgery was 46.75 ± 15.2 years and the mean BMI was 26.92 ± 5.7 kg/m^2^. Fifty percent (12 of 24) of patients were female. The right ankle was involved in 62.5% (15 of 24) of patients. The mean defect size was 6.95 ± 2.9 mm^2^. Study demographics are shown in Table 1.

### 3.3. Outcomes of Interest

The mean surgical duration was 112 ± 20 min while the mean hospitalization length was 5.58 ± 1.7 days. At last follow-up, the AOFAS increased by 27.8 points (*p* < 0.0001), while the FFI reduced of 25.3 points (*p* < 0.0001). The MOCART score was conducted on 23 patients and increased by 28.33 points (*p* < 0.0001). No complications were observed. The multivariate analysis evidenced any statistically significant correlation between data at baseline (age, gender, side, size of defect, BMI, duration of symptoms, surgical duration, hospitalization length, MOCART, AOFAS and FFI) and these at last follow-up (MOCART, AOFAS and FFI). The results of MOCART, AOFAS and FFI are shown in detail in Table 2. 

## 4. Discussion

According to the main results of the present study, AMIC for focal osteochondral defect of the talar shoulder demonstrated significant improvement in MOCART, AOFAS, and FFI at approximately two-years follow-up. No association was evidenced between patient demographic, defect size, clinical score at baseline, hospitalization, or surgical duration with the AOFAS, FFI or MOCART scores at last follow-up. No complications were observed.

A previous report on the AMIC procedure using I/III collagen membrane through malleolar osteotomy reported comparable results [20,21,22,23]. The AMIC procedure is also commonly performed for knee chondral defect. Valderrabano et al. [22] in 2013 first performed AMIC on the ankles of 26 patients, without reporting complications over a minimum of 24 months follow-up. Gottschalk et al. [21] conducted a five-year prospective cohort study on 21 patients undergoing AMIC. They found that the greatest improvement of the FFI score was evidenced within the first year, while minimal improvements were noticeable between 2 to 5 years post-operatively. Weigelt et al. [23] performed a retrospective study over 33 patients. They demonstrated a positive trend of the AOFAS and the visual analogic scale (VAS) until the fifth post-operative year, while afterwards these scores did not further increase. Further studies with longer follow-up are required to clarify the long-term outcomes of AMIC. 

Although several surgical approaches have been addressed to treat OLT, there is still no consensus on the best treatment choice [24]. Autologous chondrocyte implantation (ACI), osteochondral autograft transfer (OAT, mosaicplasty), and bulk osteochondral allograft transplantation have been addressed to OLT [25,26,27] as well innovative techniques that exploit the potential of innovative cell therapies [2]. However, these procedures sacrifice the non-weightbearing healthy cartilage, require multiple surgical interventions, and given the autologous nature of the graft, availability is limited. Furthermore, they are not readily available in many countries because of juridical issues, and given the high costs of these procedures, are often only partially covered by health insurance [28,29]. AMIC is performed in a single surgical session and does not require donor site or tissue or cell transplantation, reducing surgical time and morbidity. Moreover, compared to other procedures (e.g., ACI) with in vitro-cultured chondrocytes, the use of the membrane results in significantly-lower selling. AMIC can be performed using an arthroscopic or open technique [30,31], whereas a malleolar osteotomy usually results in optimal exposure of the talar dome and may expose patients to bony complications [32]. Malleolar osteotomy is recommended when required for adequate access to the lesion, otherwise arthroscopic or mini-open arthrotomy is recommended [22]. Recently, Galla et al. [20] published their case series of AMIC without malleolar osteotomy. At a mean of 30 months follow-up, they reported significant decrease of VAS and FFI. One patient had a transient postoperative irritation of the deep peroneal nerve and another patient had painful arthrofibrosis and underwent revision. A further patient complained of persistent pain requiring revision. 

Over the past few decades, biomedical research has focused on restoration of articular surface using scaffold implantations for the management of OLT [33,34,35,36,37]. ChondroGide was the first-used membrane for AMIC and still remains the most popular [38]. Chondro-Gide is allogenic porcine collagen I/III membrane that has been approved by the European community for cartilage reconstruction. However, ChondroGide scaffold cannot balance chondrogenesis and osteogenesis simultaneously [39]. Recently, Albano et al. [40] used a cell-free biomimetic scaffold with different layers that mimic the structures of osteochondral tissues, reporting a 31% (5 of 16 patients) failure at 30 months follow-up. 

The MOCART score is commonly used to evaluate AMIC in patients with OCL. We were unable to find any association between data at baseline and MOCART at last follow-up. The correlation between MOCART score and the clinical and functional outcomes remains controversial [41,42,43]. The MOCART score was originally designed to assess knee chondral defect, where the cartilage layer is thicker and joint space is larger than in talus. Delayed gadolinium-enhanced MRI, as well as T1 rho imaging, T2 mapping, diffusion-weighted imaging, and diffusion tensor imaging, may be more appropriate to assess cartilage status [44,45,46,47,48]. 

This study has certainly limitations. First, the small number of patients included. Two patients were secondary and tertiary referrals to the main author. Given the lack of previous documentation, it was not possible to clearly state the nature of the previous treatments. Moreover, we cannot be sure whether all the patients reported all the treatments which they were subjected to before deciding to undergo surgery. In this respect, therefore, the results are not fully generalizable. The unblinded design of the study and the lack of control group are further limitations. A randomized controlled trial comparing two or more techniques could be planned to demonstrate which one offers superior outcomes. We are aware that the high rate of patients lost during the follow-up biased our results. The hospital, in which operations were performed, is a well-known center for orthopedic surgery, attracting patients from distant areas. As a result, many patients did not agree to take a long trip to undergo an MRI for research purposes. The effect of smoking in musculoskeletal medicine has been associated with increased joint disease activity, poor functional outcomes, and poor therapeutic response [49]. The present study did not evaluate the effect of tobacco consumption on the surgical outcome, thus representing a limitation. The role of tobacco consumption is controversial. Galla et al. [20] evidence a negative association between tobacco and surgical outcome, while Wiewiorski et al. [31] showed no difference between smokers and non-smokers. All these limitations considerably affect the reliability of our results; therefore, data must be interpreted with caution. Future studies should implement these limitations performing high-quality analysis on a larger population. 

## 5. Conclusions

AMIC for focal chondral defect of the talar shoulder demonstrated clinically-relevant improvement in MOCART, AOFAS, and FFI at approximately two-years follow-up. No association was evidenced between patient demographic, defect size, clinical score at baseline, hospitalization or surgical duration with the AOFAS, FFI or MOCART scores at last follow-up. No complications were observed. 

## Figures and Tables

**Table 1:** Demographic data of the patients on admission.

Endpoint	Value at Baseline
Number of procedures	24
Mean age	46.8 ± 14.8
Female gender	50% (12/24)
Right side	62.5% (15/24)
Mean BMI	26.92 ± 5.7
Mean defect size (mm^2^)	6.95 ± 2.9
Mean duration of prior symptoms (months)	24.77 ± 32.1
Mean length of follow up (months)	25.17 ± 13.1

**Table 2:** Results of American orthopedic foot and ankle score (AOFAS) and foot-function index (FFI) at last follow-up.

Endpoint	Pre-OP	Post-OP	MD	*p*
MOCART	39.29 ± 19.4	67.62 ± 19.7	28.33	<0.0001
AOFAS	61.83 ± 15.6	80.58 ± 15.8	19.28	<0.0001
FFI	47.8 ± 19.4	22.5 ± 21.6	−25.3	<0.0001

## References

[1] Uozumi H., Sugita T., Aizawa T., Takahashi A., Ohnuma M., Itoi E. (2009). Histologic findings and possible causes of osteochondritis dissecans of the knee. Am. J. Sports Med..

[2] Migliorini F., Berton A., Salvatore G., Candela V., Khan W., Longo U.G., Denaro V. (2020). Autologous Chondrocyte Implantation and Mesenchymal Stem Cells for the Treatments of Chondral Defects of The Knee- A Systematic Review. Curr. Stem Cell Res. Ther..

[3] Verhagen R.A., Struijs P.A., Bossuyt P.M., van Dijk C.N. (2003). Systematic review of treatment strategies for osteochondral defects of the talar dome. Foot Ankle Clin..

[4] Abu-Shakra M., Buskila D., Shoenfeld Y. (2003). Osteonecrosis in patients with SLE. Clin. Rev. Allergy Immunol..

[5] Van Dijk C.N., Reilingh M.L., Zengerink M., van Bergen C.J. (2010). Osteochondral defects in the ankle: Why painful?. Knee Surg. Sports Traumatol. Arthrosc..

[6] Korner D., Gueorguiev B., Niemeyer P., Bangert Y., Zinser W., Aurich M., Walther M., Becher C., Ateschrang A., Schroter S. (2017). Parameters influencing complaints and joint function in patients with osteochondral lesions of the ankle-an investigation based on data from the German Cartilage Registry (KnorpelRegister DGOU). Arch. Orthop. Trauma Surg..

[7] Bruns J., Habermann C.R., Petersen J.P. (2016). Osteochondritis Dissecans of the Talus—A Critical Review. Z. Orthop. Unfall..

[8] Vannini F., Costa G.G., Caravelli S., Pagliazzi G., Mosca M. (2016). Treatment of osteochondral lesions of the talus in athletes: What is the evidence?. Joints.

[9] Pettine K.A., Morrey B.F. (1987). Osteochondral fractures of the talus. A long-term follow-up. J. Bone Jt. Surg..

[10] Amendola A., Panarella L. (2009). Osteochondral lesions: Medial versus lateral, persistent pain, cartilage restoration options and indications. Foot Ankle Clin..

[11] Dahmen J., Lambers K.T.A., Reilingh M.L., van Bergen C.J.A., Stufkens S.A.S., Kerkhoffs G. (2017). Reply to the letter to the editor: Comment on “No superior treatment for primary osteochondral defects of the talus”. Knee Surg. Sports Traumatol. Arthrosc..

[12] Zengerink M., Struijs P.A., Tol J.L., van Dijk C.N. (2010). Treatment of osteochondral lesions of the talus: A systematic review. Knee Surg. Sports Traumatol. Arthrosc..

[13] Lee Y.H., Suzer F., Thermann H. (2014). Autologous Matrix-Induced Chondrogenesis in the Knee: A Review. Cartilage.

[14] Gille J., Schuseil E., Wimmer J., Gellissen J., Schulz A.P., Behrens P. (2010). Mid-term results of Autologous Matrix-Induced Chondrogenesis for treatment of focal cartilage defects in the knee. Knee Surg. Sports Traumatol. Arthrosc..

[15] Von Elm E., Altman D.G., Egger M., Pocock S.J., Gotzsche P.C., Vandenbroucke J.P., Initiative S. (2008). The Strengthening the Reporting of Observational Studies in Epidemiology (STROBE) statement: Guidelines for reporting observational studies. J. Clin. Epidemiol..

[16] Wiewiorski M., Barg A., Valderrabano V. (2013). Autologous matrix-induced chondrogenesis in osteochondral lesions of the talus. Foot Ankle Clin..

[17] Marlovits S., Singer P., Zeller P., Mandl I., Haller J., Trattnig S. (2006). Magnetic resonance observation of cartilage repair tissue (MOCART) for the evaluation of autologous chondrocyte transplantation: Determination of interobserver variability and correlation to clinical outcome after 2 years. Eur. J. Radiol..

[18] Budiman-Mak E., Conrad K.J., Roach K.E. (1991). The Foot Function Index: A measure of foot pain and disability. J. Clin. Epidemiol..

[19] Kitaoka H.B., Alexander I.J., Adelaar R.S., Nunley J.A., Myerson M.S., Sanders M. (1994). Clinical rating systems for the ankle-hindfoot, midfoot, hallux, and lesser toes. Foot Ankle Int..

[20] Galla M., Duensing I., Kahn T.L., Barg A. (2019). Open reconstruction with autologous spongiosa grafts and matrix-induced chondrogenesis for osteochondral lesions of the talus can be performed without medial malleolar osteotomy. Knee Surg. Sports Traumatol. Arthrosc..

[21] Gottschalk O., Altenberger S., Baumbach S., Kriegelstein S., Dreyer F., Mehlhorn A., Horterer H., Topfer A., Roser A., Walther M. (2017). Functional Medium-Term Results After Autologous Matrix-Induced Chondrogenesis for Osteochondral Lesions of the Talus: A 5-Year Prospective Cohort Study. J. Foot Ankle Surg..

[22] Valderrabano V., Miska M., Leumann A., Wiewiorski M. (2013). Reconstruction of osteochondral lesions of the talus with autologous spongiosa grafts and autologous matrix-induced chondrogenesis. Am. J. Sports Med..

[23] Weigelt L., Hartmann R., Pfirrmann C., Espinosa N., Wirth S.H. (2019). Autologous Matrix-Induced Chondrogenesis for Osteochondral Lesions of the Talus: A Clinical and Radiological 2- to 8-Year Follow-up Study. Am. J. Sports Med..

[24] Oussedik S., Tsitskaris K., Parker D. (2015). Treatment of articular cartilage lesions of the knee by microfracture or autologous chondrocyte implantation: A systematic review. Arthroscopy.

[25] VanTienderen R.J., Dunn J.C., Kusnezov N., Orr J.D. (2017). Osteochondral Allograft Transfer for Treatment of Osteochondral Lesions of the Talus: A Systematic Review. Arthroscopy.

[26] Hangody L. (2003). The mosaicplasty technique for osteochondral lesions of the talus. Foot Ankle Clin..

[27] Imhoff A.B., Paul J., Ottinger B., Wortler K., Lammle L., Spang J., Hinterwimmer S. (2011). Osteochondral transplantation of the talus: Long-term clinical and magnetic resonance imaging evaluation. Am. J. Sports Med..

[28] Raikin S.M. (2009). Fresh osteochondral allografts for large-volume cystic osteochondral defects of the talus. J. Bone Jt. Surg. Am..

[29] Clar C., Cummins E., McIntyre L., Thomas S., Lamb J., Bain L., Jobanputra P., Waugh N. (2005). Clinical and cost-effectiveness of autologous chondrocyte implantation for cartilage defects in knee joints: Systematic review and economic evaluation. Health Technol. Assess..

[30] Usuelli F.G., de Girolamo L., Grassi M., D’Ambrosi R., Montrasio U.A., Boga M. (2015). All-Arthroscopic Autologous Matrix-Induced Chondrogenesis for the Treatment of Osteochondral Lesions of the Talus. Arthrosc. Tech..

[31] Wiewiorski M., Werner L., Paul J., Anderson A.E., Barg A., Valderrabano V. (2016). Sports Activity After Reconstruction of Osteochondral Lesions of the Talus With Autologous Spongiosa Grafts and Autologous Matrix-Induced Chondrogenesis. Am. J. Sports Med..

[32] Barg A., Pagenstert G., Leumann A., Valderrabano V. (2013). Malleolar osteotomy—Osteotomy as approach. Orthopade.

[33] Perdisa F., Filardo G., Sessa A., Busacca M., Zaffagnini S., Marcacci M., Kon E. (2017). One-Step Treatment for Patellar Cartilage Defects With a Cell-Free Osteochondral Scaffold: A Prospective Clinical and MRI Evaluation. Am. J. Sports Med..

[34] Kon E., Delcogliano M., Filardo G., Pressato D., Busacca M., Grigolo B., Desando G., Marcacci M. (2010). A novel nano-composite multi-layered biomaterial for treatment of osteochondral lesions: Technique note and an early stability pilot clinical trial. Injury.

[35] Kon E., Delcogliano M., Filardo G., Fini M., Giavaresi G., Francioli S., Martin I., Pressato D., Arcangeli E., Quarto R. (2010). Orderly osteochondral regeneration in a sheep model using a novel nano-composite multilayered biomaterial. J. Orthop. Res..

[36] Filardo G., Kon E., Berruto M., Di Martino A., Patella S., Marcheggiani Muccioli G.M., Zaffagnini S., Marcacci M. (2012). Arthroscopic second generation autologous chondrocytes implantation associated with bone grafting for the treatment of knee osteochondritis dissecans: Results at 6 years. Knee.

[37] Filardo G., Kon E., Andriolo L., Di Martino A., Zaffagnini S., Marcacci M. (2014). Treatment of “patellofemoral” cartilage lesions with matrix-assisted autologous chondrocyte transplantation: A comparison of patellar and trochlear lesions. Am. J. Sports Med..

[38] Behrens P., Ehlers E.M., Kochermann K.U., Rohwedel J., Russlies M., Plotz W. (1999). New therapy procedure for localized cartilage defects. Encouraging results with autologous chondrocyte implantation. MMW Fortschr. Med..

[39] Li X., Ding J., Wang J., Zhuang X., Chen X. (2015). Biomimetic biphasic scaffolds for osteochondral defect repair. Regen. Biomater..

[40] Albano D., Martinelli N., Bianchi A., Messina C., Malerba F., Sconfienza L.M. (2017). Clinical and imaging outcome of osteochondral lesions of the talus treated using autologous matrix-induced chondrogenesis technique with a biomimetic scaffold. BMC Musculoskelet. Disord..

[41] Kubosch E.J., Erdle B., Izadpanah K., Kubosch D., Uhl M., Sudkamp N.P., Niemeyer P. (2016). Clinical outcome and T2 assessment following autologous matrix-induced chondrogenesis in osteochondral lesions of the talus. Int. Orthop..

[42] Lee K.T., Choi Y.S., Lee Y.K., Cha S.D., Koo H.M. (2011). Comparison of MRI and arthroscopy in modified MOCART scoring system after autologous chondrocyte implantation for osteochondral lesion of the talus. Orthopedics.

[43] Aurich M., Bedi H.S., Smith P.J., Rolauffs B., Muckley T., Clayton J., Blackney M. (2011). Arthroscopic treatment of osteochondral lesions of the ankle with matrix-associated chondrocyte implantation: Early clinical and magnetic resonance imaging results. Am. J. Sports Med..

[44] Trattnig S., Ohel K., Mlynarik V., Juras V., Zbyn S., Korner A. (2015). Morphological and compositional monitoring of a new cell-free cartilage repair hydrogel technology—GelrinC by MR using semi-quantitative MOCART scoring and quantitative T2 index and new zonal T2 index calculation. Osteoarthr. Cartil..

[45] Samosky J.T., Burstein D., Eric Grimson W., Howe R., Martin S., Gray M.L. (2005). Spatially-localized correlation of dGEMRIC-measured GAG distribution and mechanical stiffness in the human tibial plateau. J. Orthop. Res..

[46] Potter H.G., Black B.R., Chong le R. (2009). New techniques in articular cartilage imaging. Clin. Sports Med..

[47] Ukai T., Sato M., Yamashita T., Imai Y., Mitani G., Takagaki T., Serigano K., Mochida J. (2015). Diffusion tensor imaging can detect the early stages of cartilage damage: A comparison study. BMC Musculoskelet. Disord..

[48] Kretzschmar M., Bieri O., Miska M., Wiewiorski M., Hainc N., Valderrabano V., Studler U. (2015). Characterization of the collagen component of cartilage repair tissue of the talus with quantitative MRI: Comparison of T2 relaxation time measurements with a diffusion-weighted double-echo steady-state sequence (dwDESS). Eur. Radiol..

[49] Al-Bashaireh A.M., Haddad L.G., Weaver M., Kelly D.L., Chengguo X., Yoon S. (2018). The Effect of Tobacco Smoking on Musculoskeletal Health: A Systematic Review. J. Environ. Public Health.

